# The dynamics of cable bacteria colonization in surface sediments: a 2D view

**DOI:** 10.1038/s41598-021-86365-1

**Published:** 2021-03-30

**Authors:** Hang Yin, Robert C. Aller, Qingzhi Zhu, Josephine Y. Aller

**Affiliations:** School of Marine and Atmospheric Sciences, Stony Brook University, Stony Brook, NY 11794-5000 USA

**Keywords:** Biogeochemistry, Marine chemistry, Biogeochemistry

## Abstract

Cable bacteria that are capable of transporting electrons on centimeter scales have been found in a variety of sediment types, where their activity can strongly influence diagenetic reactions and elemental cycling. In this study, the patterns of spatial and temporal colonization of surficial sediment by cable bacteria were revealed in two-dimensions by planar pH and H_2_S optical sensors for the first time. The characteristic sediment surface pH maximum zones begin to develop from isolated micro-regions and spread horizontally within 5 days, with lateral spreading rates from 0.3 to ~ 1.2 cm day^−1^. Electrogenic anodic zones in the anoxic sediments are characterized by low pH, and the coupled pH minima also expand with time. H_2_S heterogeneities in accordance with electrogenic colonization are also observed. Cable bacteria cell abundance in oxic surface sediment (0–0.25 cm) kept almost constant during the colonization period; however, subsurface cell abundance apparently increased as electrogenic activity expanded across the entire surface. Changes in cell abundance are consistent with filament coiling and growth in the anodic zone (i.e., cathodic snorkels). The spreading mechanism for the sediment pH–H_2_S fingerprints and the cable bacteria abundance dynamics suggest that once favorable microenvironments are established, filamentous cable bacteria aggregate or locally activate electrogenic metabolism. Different development dynamics in otherwise similar sediment suggests that the accessibility of reductant (e.g., dissolved phase sulfide) is critical in controlling the growth of cable bacteria.

## Introduction

In all previous studies, the details of cable bacteria colonization and growth in sediment have been based on 1D examination of their activity using microelectrodes. However, the lateral spatial and temporal patterns of the colonization have not been documented. In this study, we took advantage of 2D optical sensors^1,2^ to study cable bacteria colonization and activity dynamics for the first time.

The capacity for long distance electron transport by cable bacteria has been recognized as a sedimentary reaction mode by which redox half-reactions can be spatially separated while coupled through multi-cellular, electron conducting filaments^3,4^. The now classic long-distance electron transport reaction model, termed electrogenic metabolism, envisions the separation of sulfide oxidation into half reactions connected by vertically-oriented, biogenic electron transport chains^4^. In the oxic surficial sediment, where pH elevations are observed, oxygen or nitrate/nitrite are reduced and protons are consumed: O_2_ + 4e^−^ + 4H^+^ → 2H_2_O; NO_3_^−^ + 2H^+^ + 2e^−^ → NO_2_^−^ + H_2_O^5^. Reduction is coupled to oxidation of sulfide in anoxic sediment, up to centimeters distance, where decreases in pH are usually observed and protons are produced: ½H_2_S + 2H_2_O → ½SO_4_^2−^ + 4e^−^ + 5H^+^^4^. Sulfide sources can be either from solid (e.g. FeS) or dissolved (e.g. H_2_S) phases. A recent model simulation demonstrated that the resulting pH pattern can be used as a reliable indicator (i.e., fingerprint), for the occurrence of electrogenic sulfide oxidation in sediments^6^.

Cable bacteria filaments are characterized by having connected rod shaped individual cells with diameters between 0.5–2 μm, lengths ~ 2–3 μm^3,7–10^, and individual filament lengths up to 7 cm^11^. Filament densities have been found to vary spatially and temporally and can reach up to 1200 m cm^−3^, the equivalent of ~ 4 $\times$ 10^8^ cells cm^−3^ based on a mean cell length of 3 μm^12^. The appearance of pH patterns indicative of cable bacteria activity in homogenized sediment can vary between one to four weeks and may be heterogeneously distributed^13^. Previous studies resolved the details of cable bacteria activity in isolated 1D vertical profiles using microelectrodes^13,14^. However, the lateral spatial and temporal patterns of the colonization and growth of cable bacteria in sediments have not been documented. In this study, the development dynamics and heterogeneity of 2D cable bacteria colonization patterns are directly revealed for the first time utilizing planar pH and H_2_S optical sensors. We define “colonization” from the perspective of the expression of net electrogenic activity as revealed by pH patterns rather than cell abundance per se. Our experimental data show that cable bacteria electrogenic activity spreads out from distinct initial “hotspots” with different rates in otherwise similar sediments while average cable bacteria abundance in the oxic surface sediment (< 0.25 cm) does not change within the counting error during the colonization period. In contrast, cellular abundance in the anoxic zone increases, implying filament coiling and net growth. The results not only demonstrate the details of colonization—activity dynamics but also show that even in homogenized laboratory microcosms without bioturbation, the metabolic expression of cable bacteria can be spatially and temporally heterogeneous, particularly during the initial stages of colonization and growth. Thus, the impact of cable bacteria on transport—reaction patterns in sediments (such as carbonate dissolution/precipitation) must be similarly dynamic and heterogeneous.

## Materials and methods

### Sediment sampling and processing

Carbonate mud from Florida Bay, USA was collected from the upper 10 cm of mudbank deposits (overlying water depth ~ 20 cm; see Wanless and Tagett^15^) in January 2019 (25.116367° N, 80.817970° W, 21 °C), August 2019 (25.03268° N, 80.68059° W, 31 °C) and October 2020 (25.17691° N, 80.4915° W, 27 °C), sealed into plastic bags and transported by plane to Stony Brook University. The sediment collected in January was stored in the dark at 4 °C for a month before incubation experiments, and the sediment collected in August 2019 and October 2020 were processed and incubated immediately. Bulk sediment was sieved through a 1-mm nylon mesh with no added water. In each series, sieved sediment was placed into duplicate 20 × 25 × 5 cm (L × H × W) microcosms with planar pH optical sensors pre-installed onto the inner surfaces. All microcosms were filled to a depth of 10 cm, overlain by continuously aerated seawater and incubated in a 12-gallon water bucket at room temperature (22 °C) in the dark (total water depth ~ 30 cm). Microcosm overlying water was changed every 3 days with filtered (5 μm) seawater (salinity ~ 25) collected off West Meadow Beach (Long Island Sound, New York, USA).

### pH dynamics measurements

The planar pH optical sensors utilized the fluorophore HPTS (8-Hydroxypyrene-1,3,6-Trisulfonic Acid) and were modified after Zhu et al.^1^. Fluorescence images were taken at 1–2 day intervals using a Canon camera (EOS Rebel T7i), after a ratiometric calibration with fluorescence emission ratio at 545 nm after 510 nm and 430 nm wavelength excitations^1^. pH data reported here are on the NBS scale.

### H_2_S dynamics measurements

Irreversible H_2_S planar sensors were made using the diphenylcarbazone-Zn complex and covered with a gas permeable silicon outer layer^2^. Both the sample and calibration strip responses were recorded using a flatbed scanner (Canoscan 8400F). Standard solutions for calibrations were made by adjusting the pH of Na_2_S standards below 4 to convert sulfide into dissolved H_2_S (H_2_S; > 99.9% conversion) in sealed cuvettes. In the results shown, 2D H_2_S sensor image data were converted to vertical profiles by horizontal averaging of vertically-oriented membranes (widths ~ 0.5 cm). Total sulfide (ΣH_2_S) profiles were calculated based on optical sensor measured H_2_S and pH distributions with apparent *pK*_*a*_ values for H_2_S (*pK*_*a1*_ = 6.5, *pK*_*a2*_ = 13.6)^16^.

### Cable bacteria enumeration

Samples for enumeration of cable bacteria abundance were collected on day 20, 26 and 43 after microcosms were set up, in sediment obtained in January 2019, using 3 ml cutoff plastic syringes inserted randomly and vertically into microcosms. After gently pulling out the subcores, the top 5 cm were subsequently sliced and preserved in 2% formaldehyde until enumeration. The lengths of filaments and number of cells were enumerated from acridine orange (AO) stained samples using epifluorescence microscopy^7^. Cable bacteria cell abundances per mass or volume were calculated based on summed filament length in a given depth interval and the averaged cell length as 2 μm. Cable bacteria filament identity was confirmed on subsamples by fluorescence in situ hybridization (FISH) with a *Desulfobulbaceae*-specific oligonucleotide probe (DSB706)^17^. Previous studies have demonstrated that compared with the FISH method, not only are the lengths of filaments made from AO stained samples greater but also fewer subsamples were required by the AO staining method to get a consistent average^7^.

## Results and discussion

**Figure 1:**
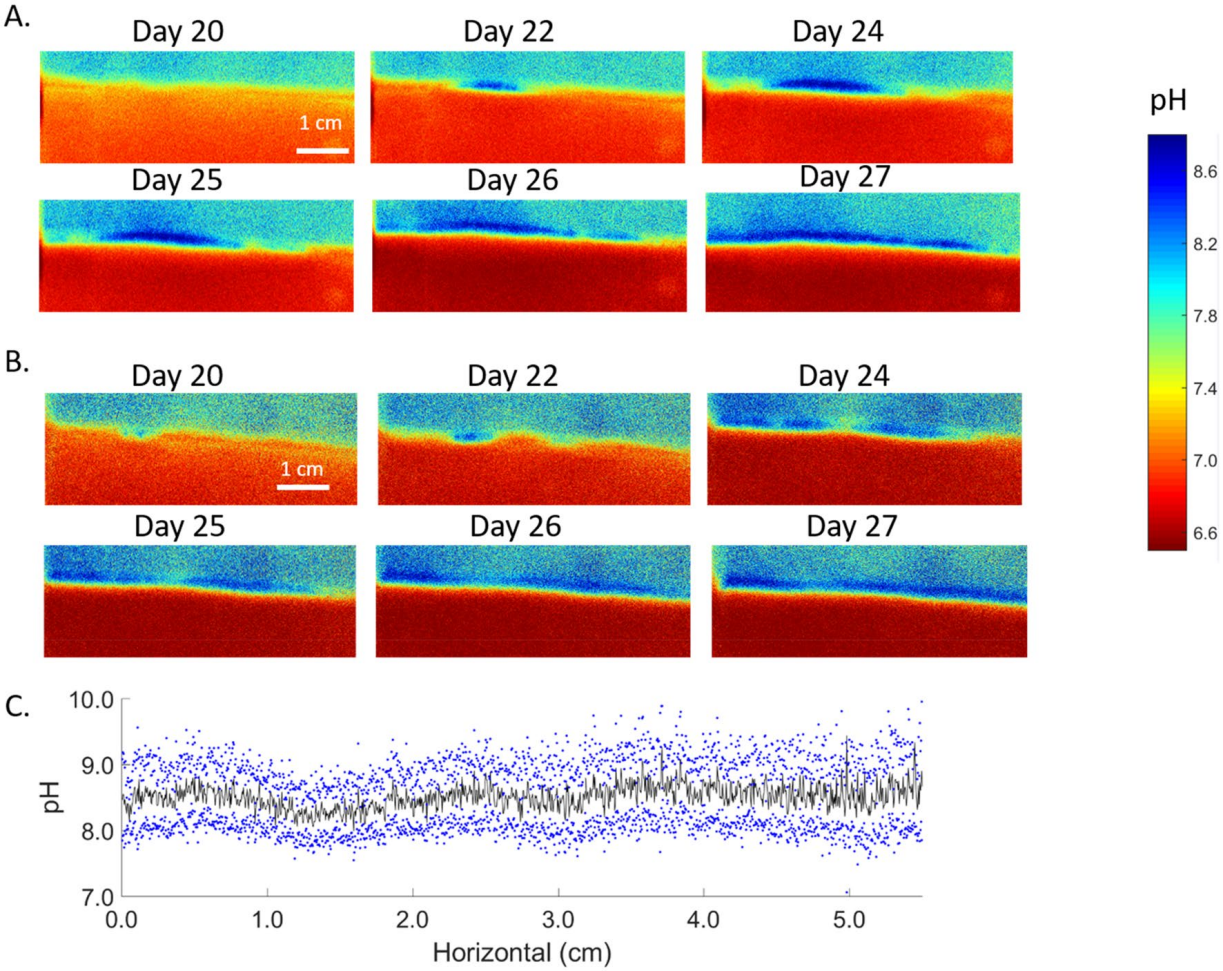
2D pH distribution dynamics of duplicate microcosms starting from day 20 (January 2019 sediment). (**A**,**B**) For duplicate microcosms, sediment surface pH maximum bands started from isolated hotspots and quickly spread across the whole surface area with spreading speed ~ 1.2 cm/day. Anoxic sediment pH decreased from day 20–26. (**C**) The horizontal pH variations within the surficial pH maximum band on day 27 (microcosm B; vertical width ~ 1 mm). The pH ± standard deviations within the band are indicated by the blue dots.

**Figure 2:**
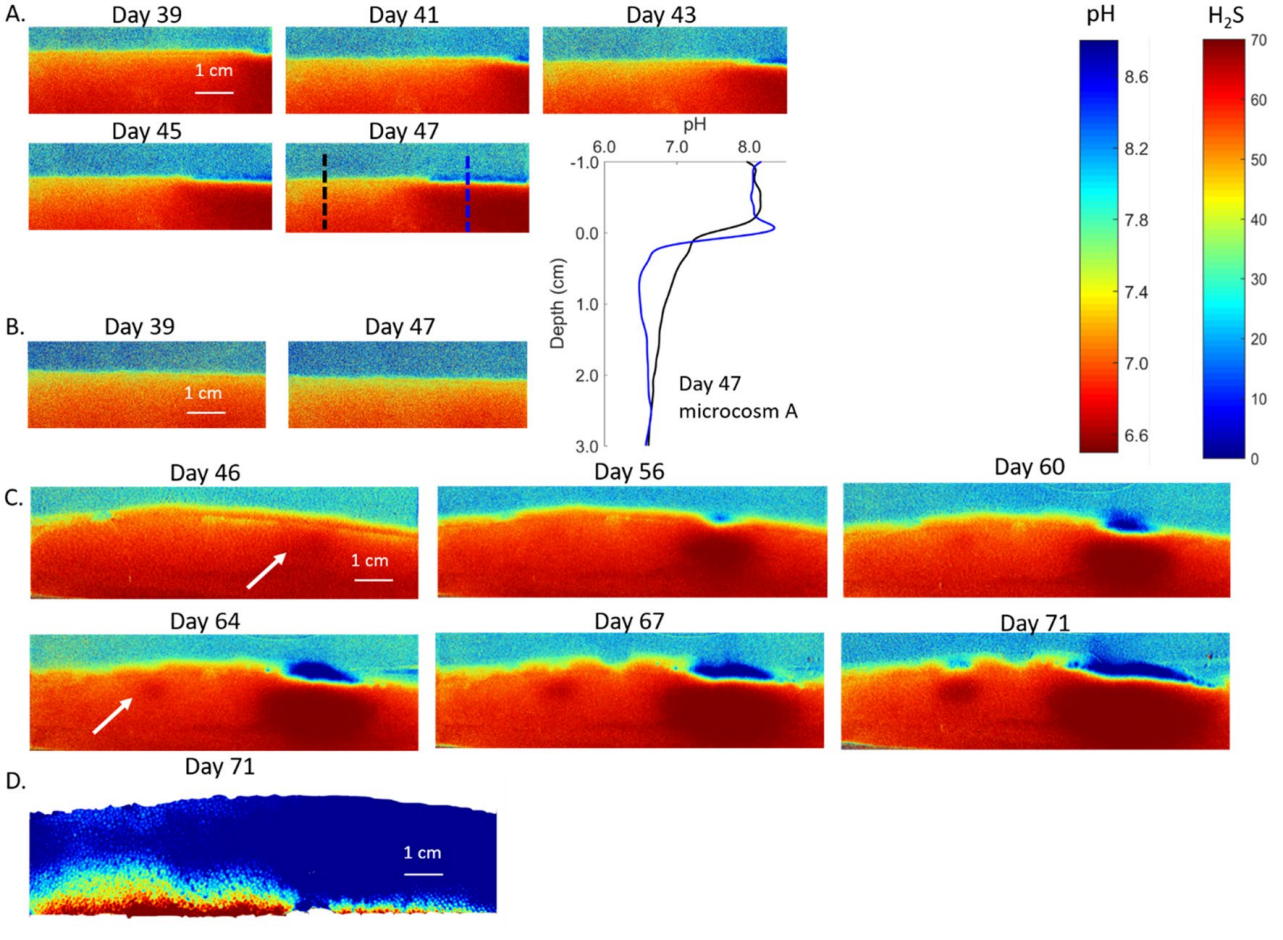
2D pH and H_2_S distribution dynamics within microcosms during colonization. (**A**) 2D pH distribution dynamics starting from day 39 (August 2019 sediment) and corresponding 1D pH profiles. The sediment surface pH maximum band started from isolated hotspots and spread across sediment surface with an average rate of 0.3 cm/day, which is much slower compared with January 2019 sediment (1.2 cm/day). The pH in the cable bacteria anodic zone is lower (blue line, A2) compared with un-colonized sediment side (black line, A1) in the pH profile panel insert. (**B**) The duplicate microcosm (August 2019 sediment) did not show sediment surface pH maxima during the same time window. (**C**) 2D pH distribution dynamics starting from day 46 (October 2020 sediment). Both surface pH maxima and deep minima expand during electrogenic colonization. The pH minima (white arrows) are evident first on day 46 and 64. (**D**) Sediment 2D H_2_S distribution on day 71 (October 2020 sediment) with upper boundary showing the sediment water interface. The H_2_S distributions are consistent with pH patterns, but the sediment H_2_S concentrations are generally lower compared with other experiment series (Fig. 4).

pH distributions within sediment microcosms showed distinct spatial and temporal patterns. For the January 2019 experiment series, the strong pH maximum bands developed in the oxic surface sediment after 20 ~ 22 days of incubation. Development was not spatially uniform. Sediment surface pH maxima started to develop from isolated points covering horizontal lengths of 0.6–1 cm at the times of imaging (Fig. 1). Within a week, the pH maximum bands expanded laterally, covering the entire 6 cm long monitoring panel, and were sustained until the end of the experiment (106 days, data not shown). The pH maximum bands were about 2 mm in vertical extent with average pH ~ 8.5. Even after the electrogenic colonization was laterally complete (day 27), the activity intensities of cable bacteria, or the impacts of their activity as reflected by the magnitudes of pH elevations and associated reactions, were still spatially heterogeneous (Fig. 1C). pH values in the underlying anoxic sediment decreased from 7.0 to 6.5 gradually as surface pH maxima formed. However, in the experiment with the sediment collected in August 2019, sediment surface pH maxima started appearing on day 39 and expanded much more slowly, with only a 2.4 cm-long lateral coverage after a week of growth in one of the duplicate microcosms and no development at all in the other (Fig. 2A,B). At the same time, where pH maxima were present, a pH minimum band developed in the anodic zone (> 2 mm depth) just below sediment surface pH maxima and expanded over time (Fig. 2A).

The October 2020 results (Fig. 2C) further resolved the cathodic and anodic zone development patterns. During colonization, the anoxic zone pH minima were evident earlier (day 46 and 64) and were distinctly wider than the sediment surface pH maxima (day 46–71). These differences in cathode and anode detection sensitivity might be caused by more rapid diffusion at the sediment–water boundary (free solution) and rapid neutralization by seawater CO_2_. They may also be related to the mode of colonization as discussed below. In addition to pH heterogeneity, the electrogenic activity also resulted in complex topographies of H_2_S distributions (Fig. 2D). In the locations where pH hotspots were found, the depths of detectable H_2_S are deeper compared with anywhere else, consistent with ongoing electrogenic sulfide oxidation metabolic activity. These data suggest that cable bacteria dynamics can be distinctly different in otherwise similar sediment (e.g. similar concentrations of dissolved H_2_S at depth), with variable development controlled by unknown factors.

**Figure 3:**
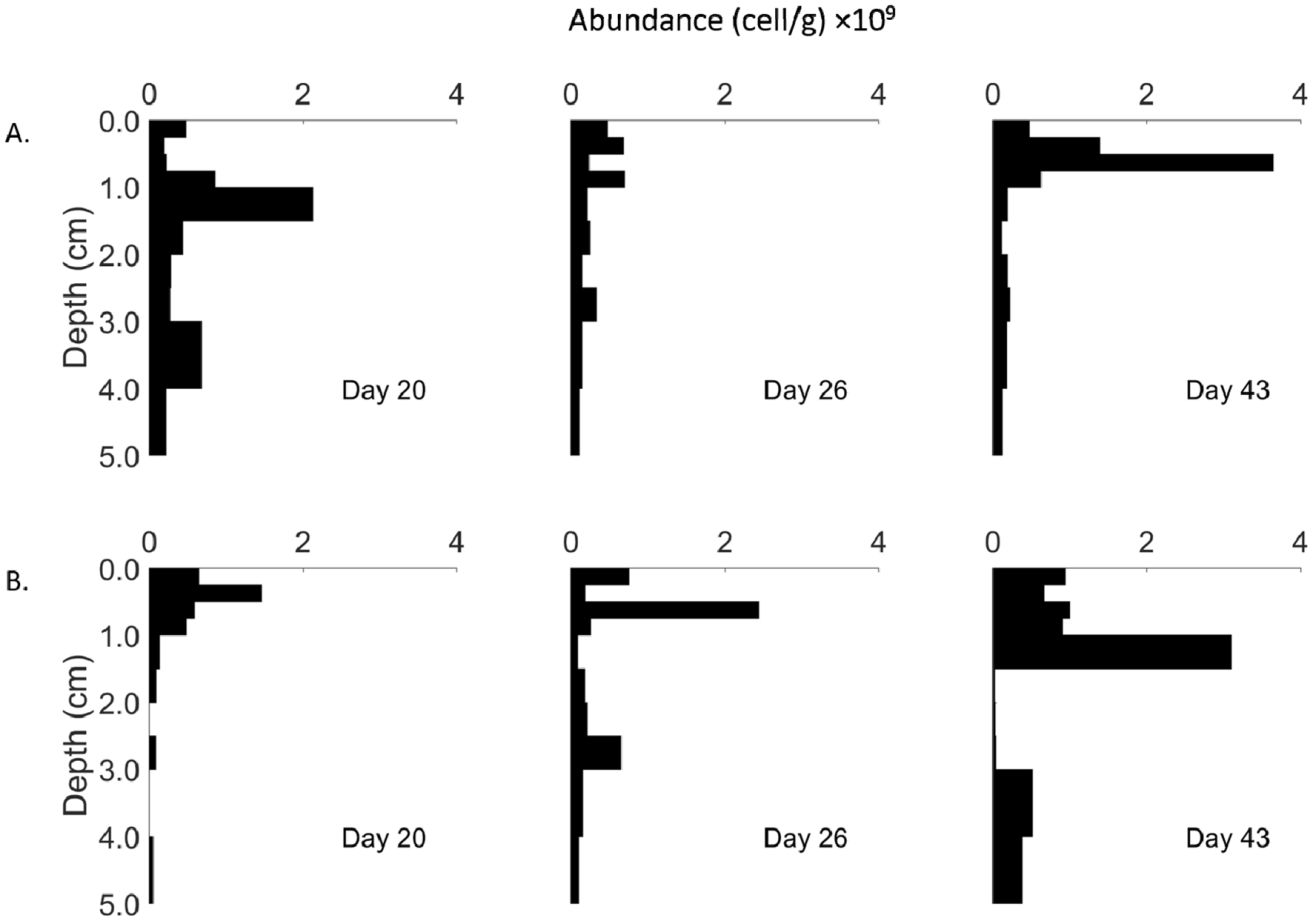
Cable bacteria cell abundance dynamics in the duplicate January 2019 sediment microcosms. (**A**) and (**B**) represent duplication microcosms. From day 20 to 43, cable bacteria can be detected throughout the top 5 cm sediment with heterogeneous abundance patterns. There were depth intervals (e.g. B 2.5–3 cm) with cable bacteria cell abundance below the detection limit of the enumeration method. The sediment surface (< 0.25 cm) cable bacteria cell abundance did not change during the monitoring period and most of the filaments were found in the cable bacteria anodic zone (0.5–1.5 cm depth).

High resolution cable bacteria abundance data are not available in this study because of the design of the experiment. However, the vertical cable bacteria abundance dynamics, which were retrieved from random locations in the January 2019 microcosms during incubation from day 20 to 43, show that cable bacteria cell abundance in the oxic zone of the sediment did not vary significantly during the primary colonization period. In contrast, one of the microcosm series (Fig. 3B) showed a distinct trend of subsurface, anodic region (0.5–1.5 cm depth) enrichment of filament cells, and importantly, both microcosm series had similar terminal distributions when electrogenic activity had expanded across the entire surface (day 43) (Fig. 3). The first time sample in series A (day 20) (Fig. 3A) is similar in abundance distribution as at day 43. It is possible that the first sample taken in series A was located in an electrogenic colonization patch, that is, locally comparable to what would become the pattern across the entire surface at day 43. These abundance data together with the high resolution pH patterns allow inference of the colonization strategy of cable bacteria, as outlined subsequently.

**Figure 4:**
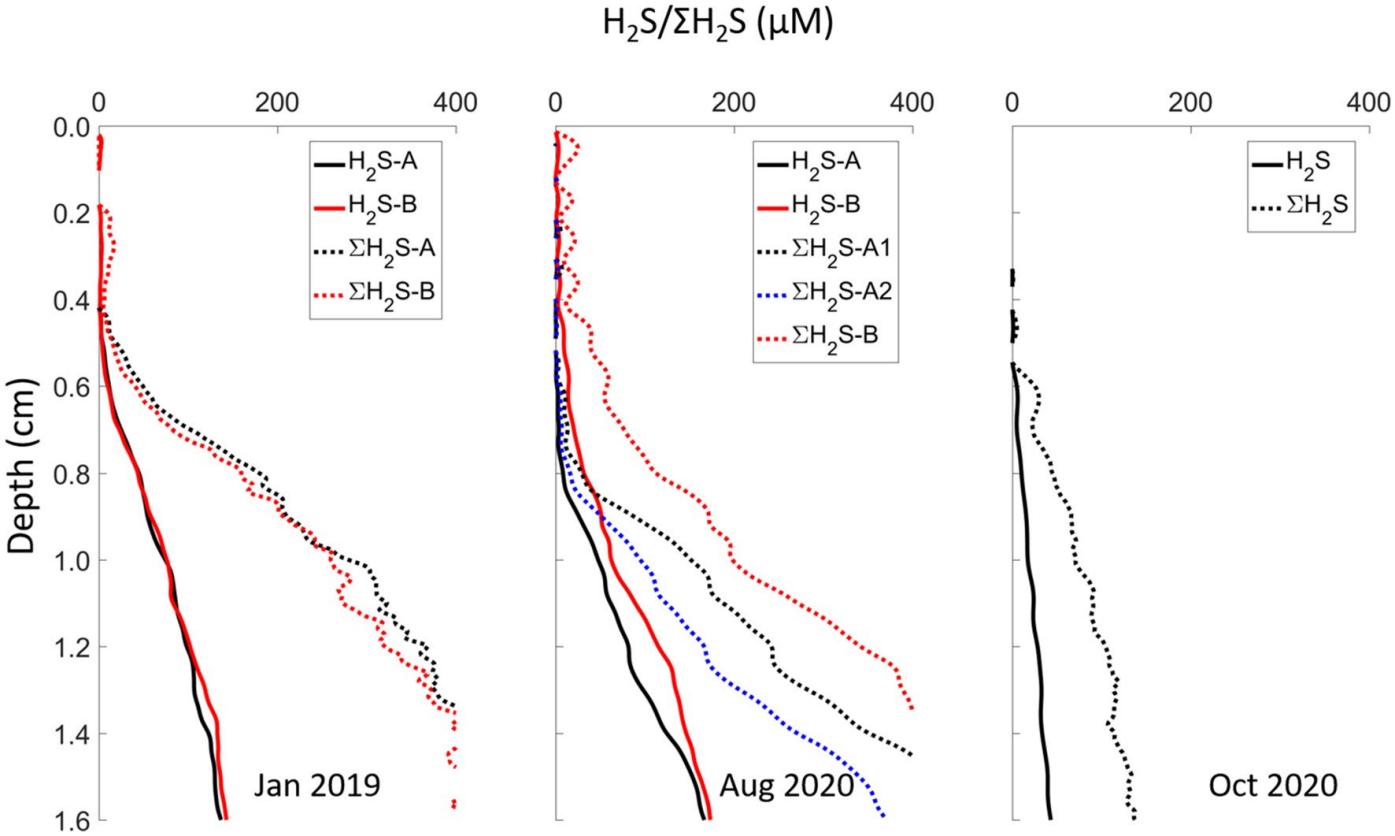
Example sediment microcosm 1D H_2_S and ΣH_2_S distributions. H_2_S and ΣH_2_S distributions were captured on day 20 in sediment obtained in January 2019 (color coding indicates duplicate microcosms; microcosm A and B are consistent with Fig. 1). H_2_S and ΣH_2_S distributions were captured on day 47 in sediment obtained in August 2019 (color coding indicates duplicate microcosms; ΣH_2_S distributions are calculated based on location specific pH vertical distributions (A1 and A2; Fig. 2 insert panel); microcosm A and B are consistent with Fig. 2). H_2_S and ΣH_2_S distributions were captured on day 53 in sediment obtained in October 2020. H_2_S detectable depths were 0.4 cm in the sediment obtained in January, 0.4–0.7 cm in the sediment obtained in August, and 0.7 cm in the sediment obtained in October. The sediment collected in October 2020 was less sulfidic than the sediments collected in January and August 2019.

**Figure 5:**
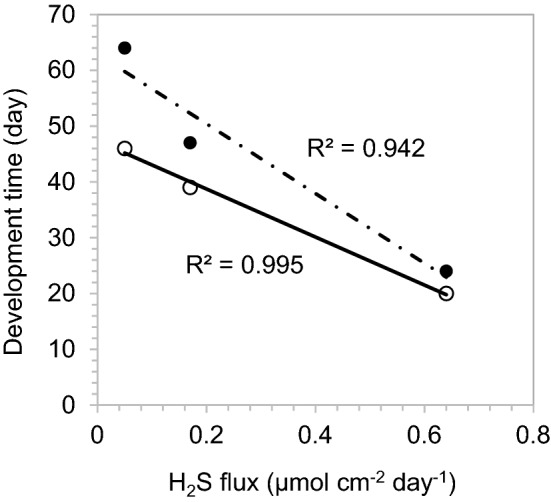
The timescale of cable bacteria colonization compared with the magnitude of H_2_S flux in microcosm sediments. The open symbol reflects the initial detection of the development of the pH maximum (or minimum) hotspot, and the solid symbol indicates the time of clear expansion of the pH maximum band across the sediment surface.

One noticeable difference among the three sets of incubation series samples was sediment porosity, which impacts the diffusive flux of H_2_S causing different cable bacteria colonization dynamics (Figs. 1 and 2) . The porosity did not vary greatly with depth: average 0.89 (January 2019), 0.65 (August 2019), 0.61 (October 2020). In these Mn/Fe-poor carbonate sediments, dissolved sulfide was the only sulfide source for electrogenic metabolism. The H_2_S flux into the anodic reaction zone is controlled by H_2_S concentration gradients, the whole sediment diffusion coefficient (D_s_) and porosity ($\varphi$). Similar H_2_S distribution profiles were observed between the January 2019 and August 2019 experimental series but it was less sulfidic for the October 2020 series (Fig. 4). Total sulfide fluxes are calculated as 0.64–0.67, 0.17–0.26 and 0.05 µmol cm^−2^ day^−1^ in the anodic zone of January, August and October sediments, respectively. The differences in H_2_S availability and flux mainly came from the diffusion coefficient and porosity variations, although gradients are also lower in the October series. Diffusive fluxes are strongly affected by porosity. According to the approximate relationship of tortuosity and diffusion coefficients (D_s_ ~ D_0_ × ${\varphi}^2$; D_0_ = free solution diffusion coefficient; φ = porosity)^18^, the diffusion coefficients in January 2019 sediment experiments were about 2 times larger than August 2019 experiments. Based on Fick’s first law (J = − $\varphi$ D_s_ × $\frac{dC}{dz}$), the diffusion rates in January 2019 sediment microcosms were about 3 times faster than the August 2019 sediment microcosms (i.e., the ratio of φ^3^ between the incubation series assuming equal concentration gradients). Unlike lithogenic sediments, where the sulfide source can come from both solid phase (FeS) and dissolved phase, lower diffusion rates of sulfide may restrict the development rates of cable bacteria when the source of sulfide is almost exclusively dissolved, such as in carbonate muds from Florida Bay. Here we found that the cable bacteria colonization time is negatively correlated with the H_2_S fluxes (Fig. 5). We also propose that the variable cable bacteria dynamics in otherwise similar sediments might have also come from seasonal differences (e.g. DOC sources) in the natural condition of cable bacteria at the different times when sediment was collected.

While the difference in the pH dynamics in the microcosm series are consistent with different sulfide fluxes, remineralization rates, and metabolic rates, they may also reflect differences in diffusive transport of reaction products and rates of neutralization reactions. For example, the low porosities of the August 2019 and October 2020 sediment may result in lower diffusion rates of protons in the sediment and enhance detection of pH minima (Fig. 2A–C), whereas maxima are located at the sediment water boundary where transport is enhanced in free solution. In contrast, the high porosity of January 2019 sediment may tend to obscure detection of proton production or consumption and also minimize differences in transport rates between the sediment–water boundary and deeper in the deposit (Fig. 1A,B).

The manner by which the sediment surface pH maxima and anoxic sediment pH minima spread, together with the cell abundance patterns with time and depth, suggests possible strategies of cable bacteria during colonization. It is known that cable bacteria can be disseminated throughout sediments and utilize multiple redox potentials, implying that they can have multiple modes of metabolism, that the electrogenic metabolic pathway is likely facultative, and that the formation of long filaments is an opportunistic morphology^7,19^. We assume that initiation of the electrogenic metabolic mode occurs randomly as a filament fragment becomes located at a stable redox interface and establishes a link between oxygen and sulfide. Once locally initiated, cable bacteria could further divide and grow, or migrate and aggregate, or both. Our data suggest that the spread of the high pH region is not associated with a large increase in bulk abundance of bacterial cells in the surface oxic layer, but do indicate that cell abundance increases in the subsurface, anoxic zone. Recently, Geerlings et al.^20^ showed that there is a labor division within individual cable bacteria filaments and that apparently only the anoxic–anodic end conserves energy. Assuming growth is largely focused into the anodic end, our cell abundance data would be consistent with a subsurface coiling of filaments^9^ rather than a strictly linear, vertical orientation, that is, a cathodic snorkel coupled to growing subsurface anodic cells.

Cable bacteria are known to be capable of moving through sediment^10^. The rapid expansion of the surface pH maximum band and deep sediment minima would also be consistent with migration of filament anodic ends toward randomly established electrogenic sites and coiling of filaments. The anoxic sediment pH minima was initially detected near the oxic sediment surface, and expanded both horizontally and vertically as a surface pH maxima enlarged (Fig. 2C), indicating the enlargement of electrogenic reaction zone in both the vertical and horizontal dimensions. A primary factor driving migration and localized growth in the initially established anodic zone may be low H_2_S concentration and high H_2_S fluxes. Once a subsurface site of decreased pH is randomly formed by cable bacteria, they may form aggregations through migration and growth in the vicinity of those hotspots. Within those hotspots, the diffusive flux of sulfide is maximum and the lower pH favors speciation of HS^-^ to H_2_S and also dissolves any FeS present, further enhancing H_2_S, which may favor the electrogenic metabolic activity of cable bacteria. Due to the expansion of the electrogenic anodic zone in the anoxic sediment, the cathodic zone, which is located in the sediment oxic layer and is characterized by pH elevation, also expands. Considering the positive feedback proposed, hotspots are ideal locations for electrogenic sulfide oxidation. The electrogenic colonization patterns we observed can also be explained by the increase of specific cell electrogenic reaction activity within the filaments already present.

The differences in development times of electrogenic activity in the different experiments demonstrate directly that there is no one timescale that defines cable bacteria establishment at a specific location in deposits, and that a wide range of colonization timescales is possible, in our cases between ~ 20 and 50 days with a direct correlation of colonization time and the estimated diffusive flux of H_2_S (Fig. 5). No matter what the colonization strategy actually used by cable bacteria (aggregation, growth of filaments, or specific cell activity stimulation within filaments), electrogenic activity shows spatially heterogeneous patterns during initiation which become more uniformly distributed as patches of cable bacteria and their activity expand both horizontally and vertically, and eventually blend together across larger areas. However, based on 2D pH patterns, cable bacteria activity, or the impacts of their activities on reaction balances, may remain spatially heterogeneous (Fig. 1C). A major implication of these electrogenic colonization patterns is that their impacts on diagenetic reaction balances and sediment–water solute fluxes are similarly highly heterogeneous and strongly time dependent.
